# Multidimensional Modeling of Biological Aging: Integrating Gait, Eye Movement, Rest‐State Functional Connectivity, and Plasma Biomarkers in non Dementia Elderly Individuals

**DOI:** 10.1002/alz.088647

**Published:** 2025-01-09

**Authors:** Jingyi Lin, Ziyu Ouyang, Tianyan Xu, Lu Shen, Bin Jiao

**Affiliations:** ^1^ Hunan International Scientific and Technological Cooperation Base of Neurodegenerative and Neurogenetic Diseases, Xiangya Hospital, Central South University, Changsha, Hunan China; ^2^ Emory University, Atlanta, GA USA; ^3^ Xiangya Hospital, Central South University, Changsha, Hunan China

## Abstract

**Background:**

Aging exhibits significant variation among individuals, with biological age as a more reliable predictor of current health status compared to chronological age. Predicting biological age is crucial for facilitating timely interventions aimed at improving the adaptation to the aging process. Given the intricate and multifactorial nature of aging, a scientific approach involves constructing a prediction model for biological age that incorporates multiple dimensions systematically.

**Method:**

This study enrolled 908 non‐dementia elderly participants, and their eye movement, gait, rest‐state functional connectivity (rs‐FC), and plasma GFAP and NfL level were measured. These parameters were used to assess an individual's biological age using logistic regression.

**Result:**

We identified 26 gait features, 2 eye‐tracking features, 19 rs‐FC features, and plasma GFAP level that exhibited significant correlations with age (p<0.05). Initially, prediction models were constructed separately using different categories of indices. Notably, gait features demonstrated robust prediction performance for age (R^2^=0.783; MAE=1.852). Subsequently, combining all indicators for the prediction model resulted in an exceptional level of performance (R^2^=0.939; MAE=1.080).

**Conclusion:**

A quantitative model of biological age was successfully constructed from a multi‐dimensional and systematic perspective, proving effectiveness in predicting an individual's biological age. This comprehensive approach, incorporating diverse indices, enhances the accuracy and reliability of age prediction models.

